# Myocardial tissue characterization by combining late gadolinium enhancement imaging and percent edema mapping: a novel T2 map-based MRI method in canine myocardial infarction

**DOI:** 10.1186/s41747-018-0037-6

**Published:** 2018-03-16

**Authors:** Pal Suranyi, Gabriel A. Elgavish, U. Joseph Schoepf, Balazs Ruzsics, Pal Kiss, Marly van Assen, Brian E. Jacobs, Brigitta C. Brott, Ada Elgavish, Akos Varga-Szemes

**Affiliations:** 1 Division of Cardiovascular Imaging, Department of Radiology and Radiological Science, Medical University of South Carolina, 25 Courtenay Dr, Charleston, SC 29425 USA; 2 Department of Biochemistry and Molecular Genetics, University of Alabama at Birmingham, MCLM 556, Birmingham, AL 35294-0005 USA; 3 Department of Cardiology, Royal Liverpool and Broadgreen University Hospital, Thomas Dr, Liverpool, L14 3LB UK; 4 University of Groningen, University Medical Center Groningen, Center for Medical Imaging - North East Netherlands, Hanzeplein 1, Groningen, 9713GZ The Netherlands; 5 Department of Medicine, Division of Cardiovascular Disease, University of Alabama at Birmingham, FOT 907, Birmingham, AL 35294-3407 USA; 6 Department of Medicine, Division of Clinical Immunology and Rheumatology, University of Alabama at Birmingham, MCLM 556, Birmingham, AL 35294-0005 USA

**Keywords:** Myocardial infarction, Myocardial edema, Myocardial hemorrhage, Magnetic resonance imaging, Tissue characterization

## Abstract

**Background:**

Assessing the extent of ischemic and reperfusion-associated myocardial injuries remains challenging with current magnetic resonance imaging (MRI) techniques. Our aim was to develop a tissue characterization mapping (TCM) technique by combining late gadolinium enhancement (LGE) with our novel percent edema mapping (PEM) approach to enable the classification of tissue represented by MRI voxels as healthy, myocardial edema (ME), necrosis, myocardial hemorrhage (MH), or scar.

**Methods:**

Six dogs underwent closed-chest myocardial infarct (MI) generation. Serial MRI scans were performed post-MI on days 3, 4, 6, 14, and 56, including T2 mapping and LGE. Dogs were sacrificed on day 4 (*n* = 4, acute MI) or day 56 (*n* = 2, chronic MI). TCMs were generated based on a voxel classification algorithm taking into account signal intensity from LGE and T2-based estimation of ME. TCM-based MI and MH were validated with post mortem triphenyl tetrazolium chloride (TTC) staining. Pearson’s correlation and Bland-Altman analyses were performed.

**Results:**

The MI, ME, and MH measured by TCM were 13.4% [25^th^–75^th^ percentile 1.6–28.8], 28.1% [2.1–37.5] and 4.3% [1.0–11.3], respectively. TCM measured higher MH and MI compared to TTC (*p* = 0.0033 and *p* = 0.0007, respectively). MH size was linearly correlated with MI size by both MRI (*r* = 0.9528, *p* < 0.0001) and TTC (*r* = 0.9625, *p* < 0.0001). MH quantification demonstrated good agreement between TCM and TTC (*r* = 0.8766, *p* < 0.0001, 2.4% overestimation by TCM). A similar correlation was observed for MI size (*r* = 0.9429, *p* < 0.0001, 6.1% overestimation by TCM).

**Conclusions:**

Preliminary results suggest that the TCM method is feasible for the in vivo localization and quantification of various MI-related tissue components.

## Key points

• TCM generates voxel-specific parametric maps based on T2 and LGE imaging

•  TCM can visualize and quantify different tissue components after MI

• TCM can be used to track the evolution of MI

• TCM differentiates between acute/chronic MI based on the presence of ME

## Background

Clinical management of myocardial infarction (MI) benefits from evaluation of the irreversibly injured necrotic area and the reversibly injured peri-infarct zone to determine the actual area at risk (AAR). The AAR consists of the MI core together with the surrounding myocardial edema (ME) and may also include myocardial hemorrage (MH), especially after revascularization [1]. The reduction of ME may serve as a novel target to minimize irreversible injury and improve left ventricle (LV) remodelling [1, 2]. MI-related ME may impede LV contraction and relaxation, induce additional necrosis, initiate potentially lethal ventricular arrhythmias, and lead to interstitial fibrosis [3–6]. Post-ischemic reperfusion further enhances the development of both intracellular and interstitial ME [7]. MH, a reperfusion injury, is frequently observed and associated with large MIs during which microvascular obstruction also occurs [8]. The presence of MH may also impact the healing process, as it is indicative of poor myocardial salvage and has an adverse effect on LV functional recovery [9, 10].

Each of these myocardial tissue changes can be characterized using different magnetic resonance imaging (MRI) approaches. Late gadolinium enhancement (LGE) imaging detects and quantifies ischemic changes of the myocardium including the expansion of extracellular space in the acute phase and evolution of fibrotic tissue in the chronic phase of MI [11]. T2-weighted MRI visualizes MI-related ME and differentiates between acute and chronic MI, as the T2 signal typically diminishes in the later phases of MI [12]; however, T2-weighted imaging is not recommended for the depiction of the AAR, mostly due to reliability concerns and the overestimation of at-risk territory [13, 14]. MH is one possible confounding factor for ME detection, as deoxihemoglobin, methemoglobin, and other blood degradation products may induce a T2 decrease in the MI core [15–17]. Indeed, T2*-weighted imaging and multi-echo T2* mapping may be used to detect and measure blood degradation products. However, none of these techniques are specific for hemorrhage, as iron overload shortens T2* and the blood oxygen-level-dependent effect may also cause subtle changes in T2* [9, 18]. In the absence of reliable ME and MH detection methods, the diagnostic and prognostic value of post-MI ME and MH quantification has not yet been exploited. High-resolution in vivo monitoring of all possible tissue types following MI, such as healthy, edematous, necrotic, hemorrhagic, or scarred tissue, would be a useful clinical tool for differentiating between acute and chronic MI and assessing the nature and extent of ischemic and reperfusion-associated injury.

Accordingly, the aim of this study was to develop a tissue characterization mapping (TCM) technique by combining the established LGE method with our novel percent edema mapping (PEM) method to enable the classification of tissue in the different MRI voxels as healthy, edematous, necrotic, hemorrhagic, or scarred using a canine model of reperfused MI.

## Methods

Our experimental protocol was approved by the Institutional Animal Care and Use Committee and was performed in compliance with the Guide for the Care and Use of Laboratory Animals of the National Institutes of Health.

### Canine preparation

Male hounds (18–20 kg, *n* = 6) were initially anesthetized with an intravenously administered mixture of ketamine (5.0 mg/kg) and diazepam (0.5 mg/kg). Following intubation, animals were mechanically ventilated (Model 2000, Hallowell EMC, Pittsfield MA, USA) and anesthesia was maintained by continuous administration of isoflurane (2.5–3% volume/volume), with intravenous fentanyl (50–100 μg every 30 min) used for analgesia. Heart rate and blood oxygen saturation were monitored and an electrocardiogram was recorded. The right femoral artery was surgically prepared and cannulated using a 6-F arterial sheath (Pinnacle, Terumo Medical Corporation, Elkton, MD, USA). Heparin was administered as needed to maintain a blood activated clotting time above 300 s. A 6-F coronary guide catheter (RunWay Kimny Mini, Boston Scientific, Natick, MA, USA) was introduced to cannulate the ostium of the left main coronary artery, and initial coronary angiography (Philips BV Pulsera, Philips Healthcare, Best, The Netherlands) was performed. A 2- to 4-mm angioplasty balloon (Maverick, Boston Scientific, Natick, MA, USA) was introduced into the left anterior descending or the left circumflex coronary artery over a guide wire, inflated, and left in position for 180 min to produce MI. After removing the balloon catheter, a second coronary angiography confirmed reperfusion. The femoral artery was decannulated, surgically ligated, and the wound was closed.

### MRI protocol

**Fig. 1:**
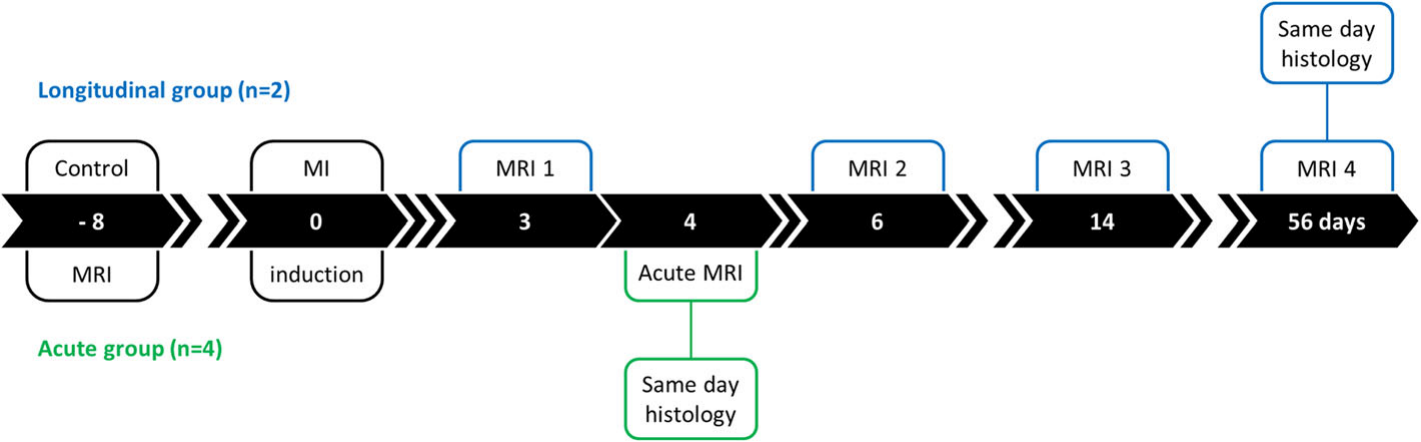
Flowchart of the study protocol. The longitudinal group (*top, blue*) underwent a baseline and four post-MI MRI scans on days 3, 6, 14, and 56. The acute group (*bottom, green*) underwent a baseline MRI as well as a single post-MI MRI on day 4

The dogs underwent cardiac MRI on a 1.5-T scanner (Signa Horizon CV/i, GE Healthcare, Milwaukee, WI, USA) before the induced MI and at various time points after MI. Four dogs were imaged and sacrificed 4 days after reperfusion to compare in vivo MRI findings in the acute phase of MI to same-day histology. Two dogs were monitored for 8 weeks to follow the evolution of MI with MRIs on days 3, 6, 14, and 56. For image acquisition, animals were anesthetetized and mechanically ventilated, as described above. Imaging was performed during breath-hold at end-inspiration. Native T2 maps and LGE images were generated for the purpose of in vivo tissue characterization at each time point. A flowchart of the protocol is shown in Fig. 1.

#### T2 mapping and T2-weighted imaging

Double inversion-recovery (black-blood) fast-spin-echo images were generated during breath-holds with varying echo times (TE). Acquisition was timed to the same end-diastolic phase of the cardiac cycle with all TEs. Six short-axis images were generated covering the entire LV with the following parameters: field of view 300 mm, image matrix 256 × 256, slice thickness 10 mm, flip angle 90°, echo train length 24, and TE 12, 20, 30, 45, 60, 75, 90, and 120 ms. The image obtained with a 60-ms TE was used as the T2-weighted image [19].

#### LGE imaging

At day 4 in the acute phase of MI, LGE imaging was performed 48 h after the administration of 0.05 mmol/kg of Gd-N-(2-butyryloxyethyl)-N’-(2-ethyloxyethyl)-N,N’-bis[N’’,N’’-bis(carboxymethyl)acetamido]-1,2-ethanediamine (Gd-ABE-DTTA), a persistent contrast agent [20, 21]. To perform LGE imaging at the other time points, 0.2 mmol/kg gadopentetate dimeglumine (Gd-DTPA, Magnevist®, Bayer HealthCare Pharmaceuticals Inc., Wayne, NJ, USA) was administered and LGE acquisition was performed 12 min after the administration of the contrast agent. LGE images were acquired using a 180°-prepared, segmented, fast gradient-echo pulse sequence in every other cardiac cycle. The inversion time was set to the optimal value to null the signal in the healthy myocardium. Identical slice orientations and positions were used for LGE and T2 maps to enable accurate co-registration of images generated by the two methods.

### Image analysis

MR images were converted to text images and analysed in ImageJ (Wayne Rasband, National Institutes of Health). Calculations were performed in either Microsoft Excel or ImageJ. Colour coding was processed in ImageJ.

#### Percent edema map

Voxel-wise R2 was calculated based on the T2 maps from the TE dependence of the signal intensity (SI) by means of a two-parameter, least-squares curve-fitting routine using the following formulas:

$$ \mathrm{SI}={\mathrm{SI}}_0\times {\mathrm{e}}^{\left(-\mathrm{TE}\times \frac{1}{\mathrm{T}2}\right)} $$

$$ \mathrm{R}2=\frac{1}{\mathrm{T}2} $$

where SI is the signal intensity and SI_0_ is the SI at the theoretical TE = 0 ms time point, also representing maximum SI.

Since post-MI myocardial R2 is in a linear relationship with the dry-to-wet weight ratio (DWR) [22], changes in tissue R2 (∆R2) can be interpreted as changes in tissue water content. ∆R2 attributable to tissue changes was calculated as follows:

$$ \Delta \mathrm{R}{2}_v=\mathrm{R}{2}_0-\mathrm{R}{2}_v $$

where R2_0_ is the R2 measured in healthy myocardium and R2_*v*_ is the observed R2 in any given myocardial voxel *v*. Thus, in healthy myocardium ∆R2_*v*_ = 0, in ME regions ∆R2_*v*_ > 0, and in regions where the water content is decreased (mature scar), ∆R2_*v*_ < 0. To determine a universal percent edema (PE) scale in terms of ∆R2_v_ values, the value PE = 0% was arbitrarily assigned to the healthy myocardium, where ∆R2_v_ = 0, corresponding to R2_0_ = 18.7±1.2 s^–1^ based on the control R2 maps generated prior to intervention. The corresponding DWR in this normal myocardium was 0.23±0.01. PE = 100% was assigned to the ∆R2 of pure water, which, by definition, corresponds to a DWR of zero. Based on the R2 of pure water (R2_H2O_ = 0.27 s^–1^), ∆R2 of pure water can be calculated as follows:

$$ \Delta \mathrm{R}{2}_{H2O}=\mathrm{R}{2}_0-\mathrm{R}{2}_{H2O}=18.7{\mathrm{s}}^{-1}-0.27{\mathrm{s}}^{-1}=18.43{\mathrm{s}}^{-1} $$

This value is the change in R2 when proceeding from healthy myocardium (DWR = 0.23) to pure water (DWR = 0). This ∆R2_H2O_ corresponds to the entire theoretical range of change in water content covering the corresponding range of PE from PE = 0% to PE = 100%, and thus is used to convert any observed R2 to the corresponding PE value. To calculate the PE values for all myocardial voxels, ∆R2_*v*_ was calculated for each voxel *v*. In this manner, the R2 map was transformed into a ∆R2 map. ∆R2_*v*_ was considered zero in all voxels where R2_*v*_ was within the range of R2_0_±2 standard deviations. Subsequently, voxel-wise PE_*v*_ values were calculated for the entire slice (percent edema per slice, PES) to generate a PEM as follows:

$$ {\mathrm{PE}}_v=\left(\Delta \mathrm{R}{2}_v\div \Delta \mathrm{R}{2}_{H2O}\right)\times 100 $$

#### LGE evaluation

Endo- and epicardial contours were manually traced, and the remote myocardium was selected using a region of interest. Further steps were automated to avoid observer bias. MI was defined as pixels that displayed an SI value above the mean SI of the remote myocardium plus sixfold the standard deviation (6 SD) [23]. MI pixels were counted, and the ratio of infarcted to total myocardial area of a given slice (percent infarct per slice, PIS) was subsequently calculated.

#### Tissue characterization map

**Fig. 2:**
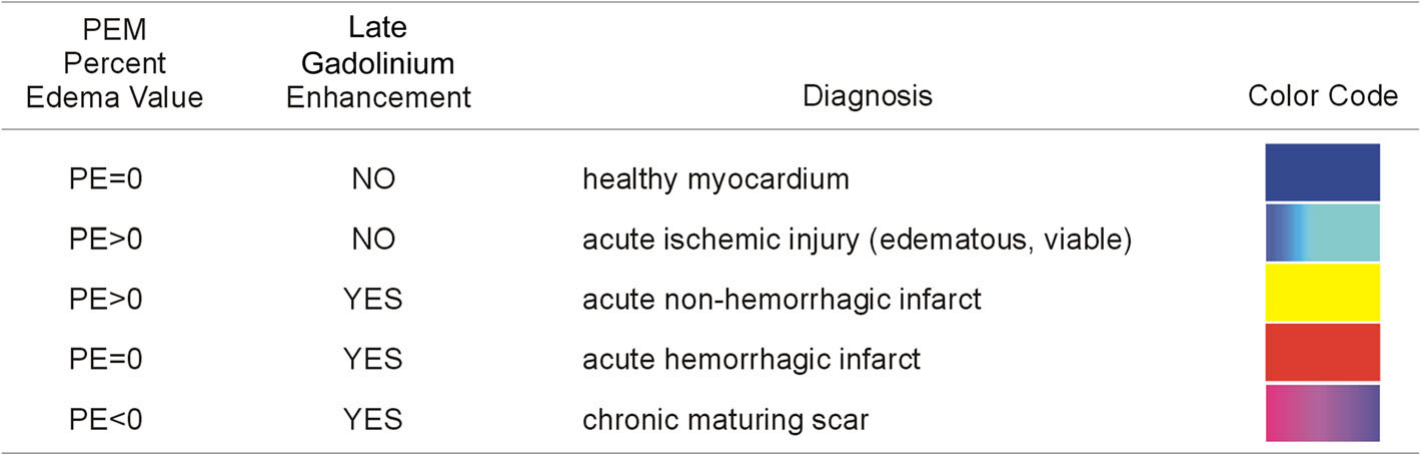
The criteria for the generation of TCM. Note that the severity of ME and the maturity of scar would be represented in the TCMs on continuous colour scales

Voxel-wise TCMs were generated by a computer routine based on criteria (Fig. 2) that combine the information from PEM and LGE images. Tissue characterization was based on the presence or absence of ME, or the presence of “negative ME”, while also taking into account voxel enhancement in the LGE image. Importantly, while “PE = 0” in healthy myocardium indicates the absence of ME, ME is present in hemorrhagic MI. Nevertheless, ME is not seen in the PEM of such hemorrhagic MI due to the cancelling effect of ∆R2 induced by methemoglobin. Since MH occurs in the center of the MI and is enhanced in LGE, it can be differentiated from healthy myocardium and quantified (percent hemorrhage per slice, PHS) using our method.

### Histopathology

**Fig. 3:**
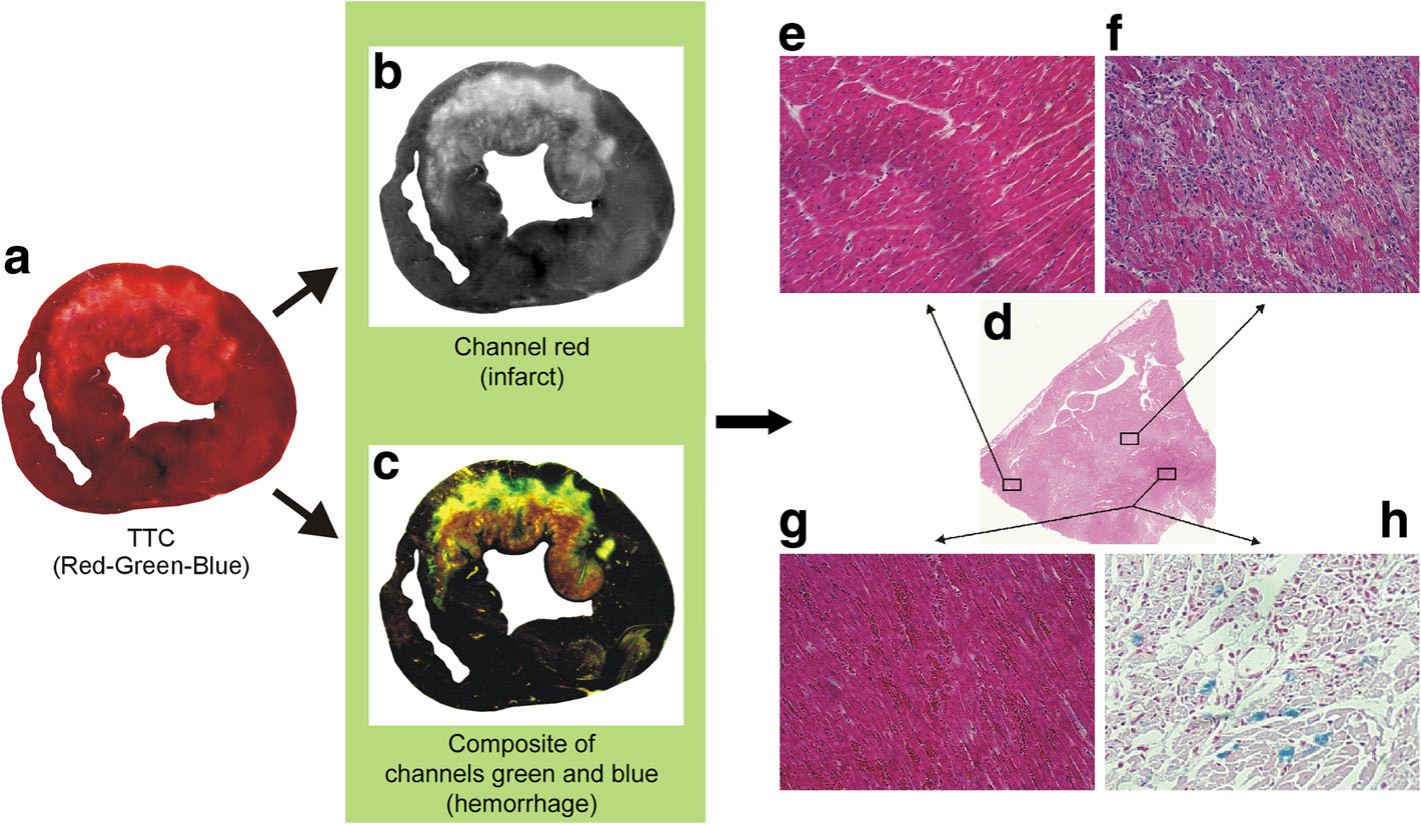
The processing steps of TTC-stained photographs (**a**) are shown. The red channel of the original TTC photo depicts MI borders most accurately (**b**). Composite of green and blue channels delineates the hemorrhagic region as a *light brown* area within the MI (**c**). Hematoxylin-eosin-stained microscopic slides (**e**–**g**) show magnified regions of the myocardial segment (**d**) in typical areas of viable tissue (**e**), non-hemorrhagic infarct (**f**), and hemorrhagic infarct (**g**). A Prussian blue-stained sample from the same hemorrhagic MI area is also shown (**h**)

Triphenyl tetrazolium chloride (TTC) staining was performed in vivo prior to inducing cardiac arrest [24]. A solution of 12.5 ml/kg of 2% TTC saline was intravenously administered and maintained in the circulation for at least 20 min [24, 25]. The animal was then euthanized with a high dose of pentobarbital followed by 100 ml of 2M potassium chloride solution. The heart was excised, rinsed with saline, and sliced in 3-mm increments using a commercial meat slicer. Both sides of each TTC slice were photographed using an Olympus C 4000 Zoom Digital camera. TTC photograph analysis was adapted from work by Ruifrok et al., in which the three colour channels (red, green, and blue) were separated in ImageJ [26]. After splitting the three channels (Fig. 3), the red channel was displayed as a greyscale image, where viable tissue is shown as dark grey and irreversibly injured regions are bright. MI borders were traced using these images. To highlight MH selectively, the blue channel image was displayed in the red channel and merged with the green channel, resulting in a composite image where hemorrhagic regions appear as light brown within the green-yellow non-hemorrhagic region. This method was further validated with hematoxylin-eosin and Prussian blue microscopic histology.

The PIS and PHS were calculated and compared with MRI data by adding MI and MH areas in three TTC slices that corresponded to one MRI slice. Values were expressed as a percentage of total LV myocardial area in that slice.

### Statistical analysis

Statistical analysis was performed using SigmaStat (version 2.03; SPSS, Inc.). A normality test was performed to determine whether the sample had a Gaussian distribution. Data with a normal distribution are reported as mean±SD. Data with a non-normal distribution are reported as median [25^th^, 75^th^ percentiles]. Student’s *t* test was used to compare data with normal distribution and equal variance, while the non-parametric Wilcoxon test was employed for non-normal distributions. Pearson’s correlation and Bland-Altman analyses were performed to compare the PIS and PHS from MRI to those obtained with TTC staining. Overestimations of results from TCM were calculated for each slice and each dog, using the TTC results as reference. Rejecting the null hypothesis at α = 0.05 with a *p* value < 0.05 indicated statistical significance.

## Results

All six animals successfully survived the MI induction and completed all assigned MRI sessions. Mean myocardial and liver T2 values prior to MI induction were 53±2.5 ms and 49.6±3.6 ms, respectively.

**Fig. 4:**
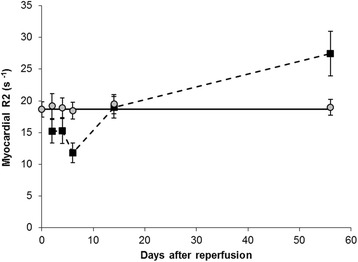
Mean (±SD) R2 values in the MI regions as delineated by LGE (*black squares*). MI R2 was significantly different from remote R2 throughout, except on day 14. Peak ME (lowest R2) was detected on day 6, and ME retreated and R2 returned to baseline by day 14. Peak R2 was detected in mature scars at 8 weeks. Remote R2 (*grey circles*) stayed constant and was not different from the baseline (*solid line*) at any of the time points over the course of 8 weeks

The evolution of R2 values over eight weeks in the longitudinal experiments is shown in Fig. 4. Compared to remote myocardium, a sustained, significant decrease in R2 in the MI region was observed throughout the first week (R2_0_ = 18.7±1.2 s^–1^; *p* < 0.0074), with the lowest MI R2 detected on day 6 (11.8±1.6 s^–1^). By day 14, ME retreated and R2 returned to baseline level, indicated by a lack of statistical difference between the R2 of the MI and remote regions. The highest R2 was detected in mature scars at 8 weeks (27.5±3.5 s^–1^).

**Fig. 5:**
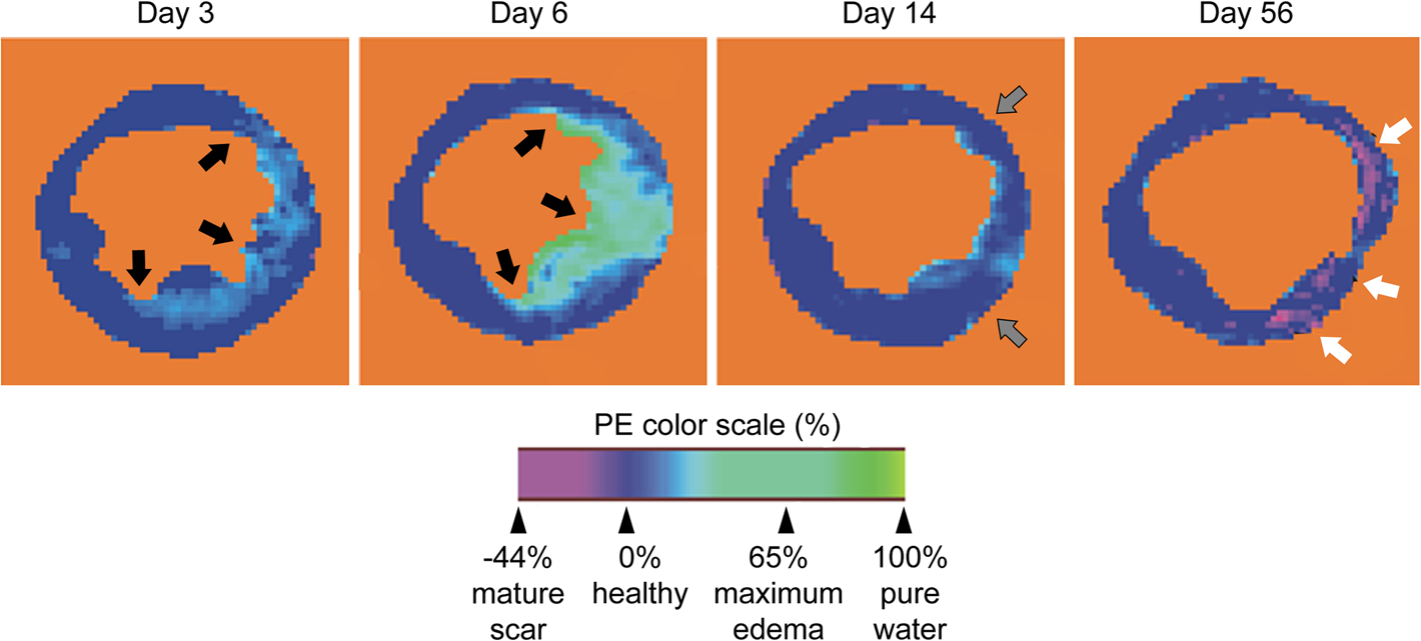
Corresponding short-axis PEMs are shown on days 3, 6, 14, and 56 following the induction of a reperfused MI in a dog. The PE values are shown on a colour scale representing varying degrees of ME and scar maturation. ME was detectable throughout the first week (*black arrows*). On day 3, ME is clearly apparent in and around the MI region, with a central clearing in the MI, representing hemorrhage. Maximum ME was detected on day 6, by which time the hemorrhage was cleared. ME was almost completely resolved by day 14. The newly formed scar tissue (*grey arrows*) had a PE value close to that of the normal myocardium. In the course of maturation, however, the scar tissue shrank and lost water, resulting in negative PE values by day 56 (*white arrows*). Note also the wall thinning over the course of the 8 weeks in these regions

Representative PEMs generated at several time points following reperfusion in a dog followed for 8 weeks are shown in Fig. 5. Maximum ME was detected on day 6, by which time most of the dead myocytes and haemorrhage had been cleared away by macrophages. By the end of the second week, ME had almost completely retreated with the exception of residual ME in a few voxels within the MI region. At 8 weeks following reperfusion, due to the maturation process of scar tissue, PE values in the scar became “negative”, indicating that their water content was lower than that of normal myocardium.

**Fig. 6:**
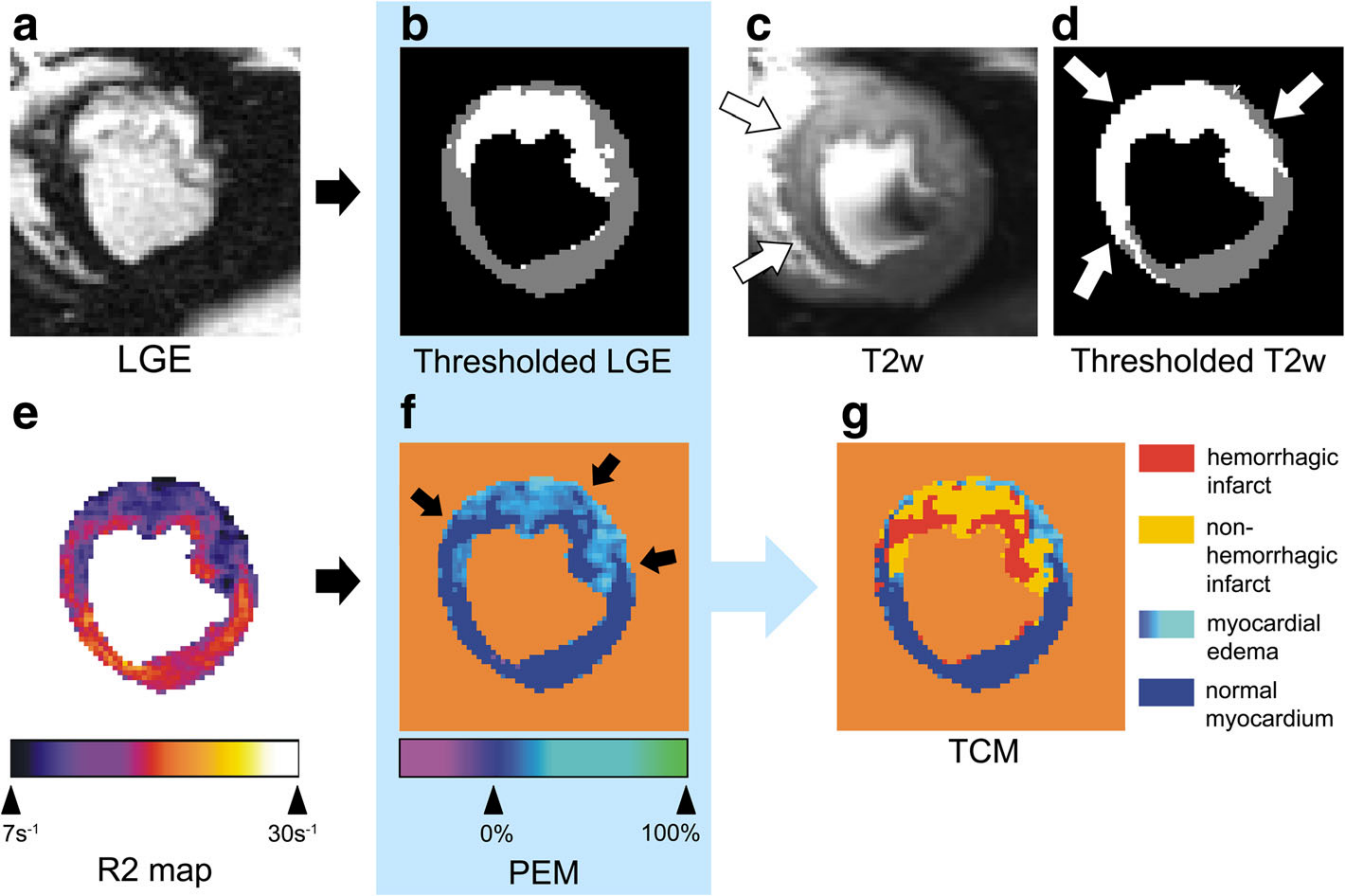
Corresponding short-axis MR images and post-processed parametric maps are shown. The MI region appears enhanced in the LGE image (**a**). Thresholded LGE image shows the irreversibly injured region as a *white area* (**b**). T2-weighted image (**c**) and corresponding thresholded T2-weighted image (**d**) highlight the increased signal in the MI region and septum (*white arrows*). Note that the crude method of thresholding T2-weighted images overestimates ME and is unable to differentiate regions with a varying extent of ME. Therefore, T2-weighted imaging cannot differentiate hemorrhagic from non-hemorrhagic MIs. PEM (**f**) calculated from the R2 map (**e**) shows the AAR (*black arrows*). There is an apparent lack of ME in the center of the MI due to cancelling effect by acute hemorrhage. TCM (**g**) generated from **b** and **f** defines the ME region surrounding the non-hemorrhagic part of the necrotic tissue, and the hemorrhagic region in the center of the MI. Corresponding TTC pathology is shown in Fig. 3

**Fig. 7:**
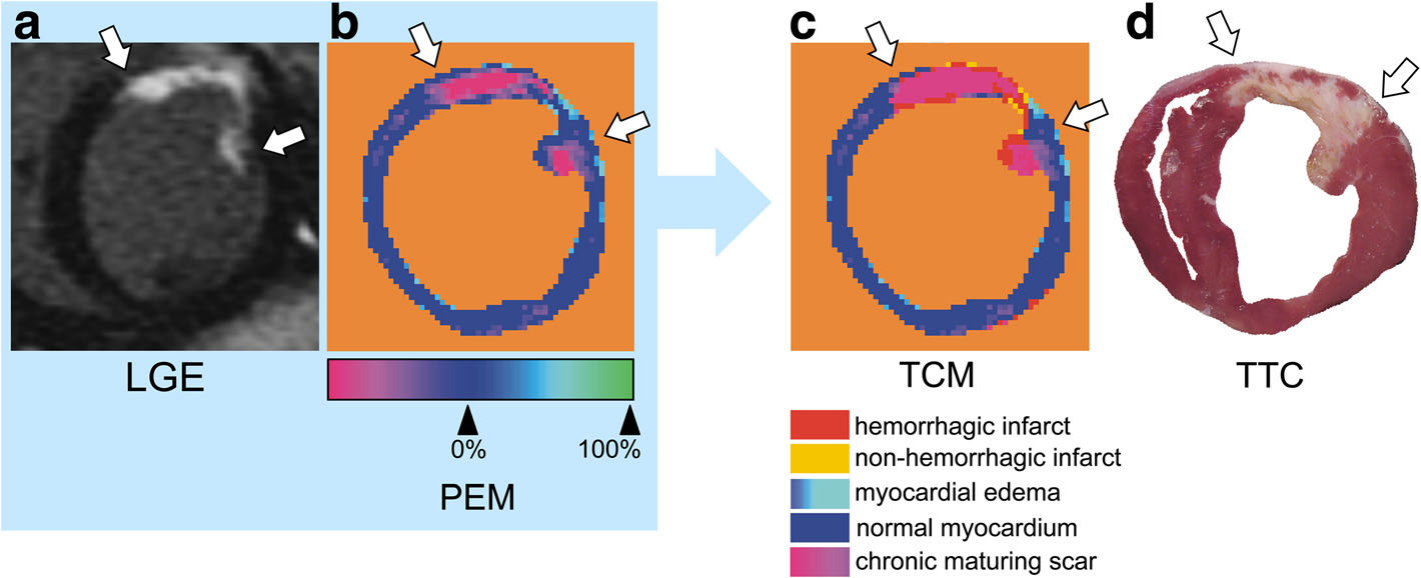
Corresponding short-axis MRI slices of LV are shown at 8 weeks after the induced reperfused MI in a dog. In the LGE image, hyper-enhancement is apparent in chronic scar (**a**, *white arrows*). PEM highlights mature scar as “negative edema” due to decreased water content (**b**). Water content varies depending on the maturity of the scar, as depicted by the gradually changing hue of the purple end of the colour scale. Virtually the entire MI (as detected by LGE) is identified by TCM as chronic scar without significant ME surrounding the infarcted region (**c**). Thus, certainly on TCM, this infarct cannot be mistaken for an acute MI. Corresponding TTC-stained photo shows mature collagenous scar without hemorrhage (**d**)

An example of a TCM generated based on the combination of PEM and LGE in a dog 4 days after a hemorrhagic acute MI is shown in Fig. 6. Another example of a TCM of a mature scar presented in a dog 8 weeks following MI reperfusion is shown in Fig. 7. TCM post-processing, including the Microsoft Excel macro and ImageJ image manipulations, took approximately 2 h per image set.

**Fig. 8:**
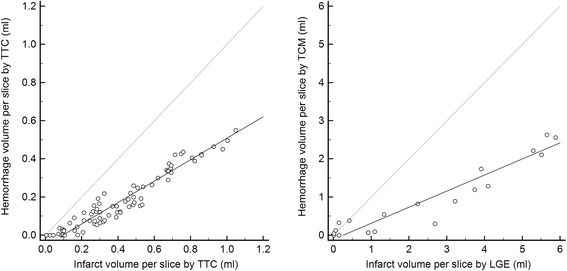
The dependence of per-slice hemorrhage volume on MI volume is shown with data taken from all infarcted TTC-stained slices of four dogs (*left panel*) and from the corresponding MRI slices of the same dogs (*right panel*). A strong linear relationship was found in both comparisons. Interestingly, the regression line has a negative *y*-intercept (*left panel*), which suggests that for MH to occur, the MI must surpass a minimum size; i.e. in very small infarcts MH is unlikely

The size of MI, ME, and MH measured by TCM and corresponding TTC is shown in Table 1. A significant difference was observed in PES measurements between T2-weighted imaging and PEM (*p* < 0.0001). TCM measured significantly higher PHS and PIS compared to TTC assessment (*p* = 0.0033 and *p* = 0.0007, respectively). A significant relationship was observed between the extent of MH within the MI region and the size of MI in dogs sacrificed 4 days after reperfusion (Fig. 8), with a correlation *r* = 0.9625 (*p* < 0.0001) and regression line of *y* = 0.54*x* – 0.05. Thus, in this particular animal model, approximately 50% of the MI tissue seems to have contained MH. A similar relationship was observed between MH and MI size obtained with in vivo MRI, with a correlation *r* = 0.9528 (*p* < 0.0001) and regression line of *y* = 0.4*x* – 0.06.

**Table 1:** MRI- and TTC-based quantification of ME, MH, and MI

	T2w	TCM	TTC	*p*
PES (%)	42.7 [4.9, 62.1]	28.1 [2.1, 37.5]		< 0.0001
PHS (%)		4.3 [1.0, 11.3]	3.5 [0.6, 5.8]	0.0033
PIS (%)		13.4 [1.6, 28.8]	9.7 [3.1, 16.0]	0.0007

Data are displayed as median [95% confidence interval]. *T2w* T2-weighted, *TCM* tissue characterization map, *TTC* triphenyl tetrazolium chloride staining, *PES* percent edema per slice, *PHS* percent hemorrhage per slice, *PIS* percent infarct per slice

**Fig. 9:**
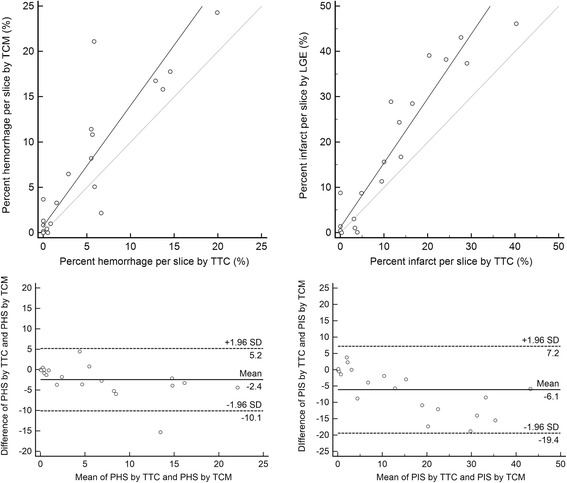
Correlation and Bland-Altman analysis between percent hemorrhage per slice (PHS) by TTC and PHS by TCM (*left panel*), as well as percent infarct per slice (PIS) by TTC and PIS by LGE (*right panel*) are shown

Figure 9 shows the correlation between PHS by TCM and PHS by TTC (*r* = 0.8766, *p* < 0.0001). Bland-Altman analysis revealed a mean overestimation of PHS by TCM of 2.4%. Similarly, a strong correlation was found between PIS by LGE and PIS by TTC (*r* = 0.9429, *p* < 0.0001), with a systematic overestimation of PIS by 6.1%.

## Discussion

This investigation sought to develop TCM, a new image post-processing approach that combines T2 mapping and LGE imaging for the characterization of post-infarct myocardium. We have shown with a preliminary set of animals that the TCM technique is able to visualize the possible five major myocardial tissue components (healthy myocardium, edema, hemorrhage, necrosis, and scar) in a single map.

Baseline myocardial R2 results in our study were in agreement with findings from previous studies [27, 28]. R2, an intrinsic parameter, is insulated from extraneous factors (field inhomogeneity, regional variations in proton density, etc.) and is linearly related to tissue water content [22]. Additionally, R2 is independent of the pulse sequence or MRI equipment used. Unlike T2-weighted signal intensity, which is inherently prone to signal inhomogeneities and erroneous estimation of the extent of ME, R2 is a reliable and reproducible parameter for quantitative parametric imaging.

The decrease in tissue R2 has been correlated with elevated tissue water content, which typically occurs with acute ischemia [22, 29]. Post-ischemic reperfusion has been shown to further enhance the development of both intracellular and interstitial edema in injured regions [7]. Subsequently, expansion of MI and progression of microvascular obstruction have been demonstrated, even after reestablishing flow to the occluded coronary artery [30]. Therefore, the clinical significance of reperfusion injury following MI has been well established [8, 31]. However, to date, no imaging modality has been able to reliably visualize and quantify the wide spectrum of tissue changes that occur during these events.

Using the PEM method, we consistently found that ME was detectable within both the MI region and neighbouring AAR within 2 weeks of MI reperfusion. In necrotic regions, and subsequently areas of granulated tissue, R2 was further decreased, reaching its lowest value approximately 5-6 days following reperfusion. Thus, when obtained in the acute phase of MI, PEMs may also prove useful in detecting and quantifying acute MI without the use of a contrast agent. Such visualization of the per-voxel severity of edema is an advantage of PEM over T2-weighted imaging, which is only able to distinguish between enhanced and non-enhanced regions. Furthermore, our results indicate that T2-weighted imaging overestimates the extent of ME, a fact that has been similarly reported from other investigations [32].

ME almost completely retreated on day 14 (R2 returned to its control level), and by the end of the eighth week, R2 in the mature scar increased to a level above its pre-ischemic control value. The increased R2 was due to scar formation, in agreement with ex vivo findings from other investigations [33, 34]. This phenomenon occurs due to progressive water loss by the maturing scar and, by the eighth week, water content is significantly lower than in normal myocardium [33]. Therefore, a confluent, mature scar appears as a “negative edema” in the PEM. The ability to differentiate scarred tissue is yet another advantage of the PEM method over T2-weighted imaging. Alongside scar maturation, thinning of the ventricular wall occurred, making the detection of such areas difficult with a single T2-weighted image. This is especially true for cases in which the overlying epicardial adipose tissue causes an SI increase in that area, potentially causing an overestimation of edema duration with T2-weighted imaging [33]. PEM was also able to visualize mature scars in chronic MI. This suggests that PEM may also be useful in differentiating between acute and chronic MI. Furthermore, the PEM method is able to detect ME in vivo. Post mortem, neither TTC staining nor traditional microscopy are able to visualize ME. Although the expansion of the interstitium seen on microscopy may be suggestive of tissue edema, it is a fairly subjective, non-quantitative method.

By combining the clinically established LGE method with our novel PEM method, we have shown the feasibility of the combined TCM technique in reperfused MI using a canine model. Our results have demonstrated good agreement with post mortem macro- and microscopic histology with only minor differences. These differences can be attributed to the different nature of the information provided by these modalities. MRI has a clear advantage over histological methods, not only for its non-invasive and in vivo applicability, but also because all tissue in a given slab contributes to the imaging results, enabling the evaluation of the entire organ. When using histological methods, only a portion of the tissue can be quantified. For example, with TTC staining, only the surface of a given slice can be visualized. With microscopy, the tissue is only sparingly sampled, and the microscopic evaluation and quantification of an entire organ would be impractical.

The excellent correlation observed between MI size and the extent of MH has been previously reported by other investigators [35]. It has been shown that the size of MH depends primarily on the duration of the coronary occlusion and the subsequent reperfusion [35]. In the present study, dogs were subjected to 180-min ischemia, during which the activated clotting time was controlled. Thus, under such standardized conditions, the correlation between MI size and MH size is not surprising.

The novel TCM method has the potential to visualize and quantify tissue changes following ischemic myocardial injury for a broad scale of tissue types (edematous, necrotic, hemorrhagic, and scarred), with a resolution governed by the spatial resolution used for the MR image acquisition itself. Importantly, the TCM method could be easily implemented with any of the currently used clinical MRI scanners. Furthermore, TCM may prove useful for the evaluation of reperfusion strategies in light of the occurrence of iatrogenic myocardial hemorrhage and for tracking iron-labelled cells, a current problem for invasive cardiologists [36]. Accurate mapping of ME also holds great potential in a variety of other diseases that affect the heart, such as myocarditis, trauma, or post-transplantation rejection [37–39]. When analyzing a TCM acquired in vivo, the in vivo “histologic” diagnosis can be made at a glance. For example, determining whether the MI is acute or chronic, hemorrhagic or non-hemorrhagic, as well as the quantification of differentiated tissue types are all evaluations afforded by in vivo TCM.

There are certain limitations to the current study that deserve special mention. First, this study included a limited number of animals due to the complexity of imaging protocols and longitudinal follow-ups. Such a small cohort only provides preliminary results regarding the feasibility of the TCM approach. Second, the TCM method in its present form relies on the LGE method, which has its own limitations. In the present study, LGE overestimated infarct size by approximately 6%. The discrepancy may have been due to partial volume effects that are an inherent problem when thresholding 10-mm-thick MRI slices to determine viability. It is also crucial that LGE imaging and R2 mapping be co-registered, which is relatively easy in an anesthetized and mechanically ventilated animal, but may be problematic in the clinical setting as consecutive breath-holds may not be equal in depth, leading to misalignment of cardiac slices. With the introduction of fast mapping protocols and respiratory-gated acquisition techniques, however, this problem may be circumvented. Finally, two different contrast agents, Gd-ABE-DTTA and gadopentetate dimeglumine, were used to image the acute and chronic phase of MI, respectively. However, it has been shown that both contrast agents provide the same MI size measurements in terms of statistical significance [40].

In conclusion, our preliminary results suggest that the TCM method is feasible for the in vivo localization and quantification of various MI-related tissue components.

## Abbreviations

AAR: Area at risk
DWR: Dry-to-wet weight ratio
Gd-ABE-DTTA: Gd-N-(2-butyryloxyethyl)-N’-(2-ethyloxyethyl)-N,N’-bis[N’’,N’’-bis(carboxymethyl)acetamido]-1,2-ethanediamine
LGE: Late gadolinium enhancement
LV: Left ventricle
ME: Myocardial edema
MH: Myocardial hemorrhage
MI: Myocardial infarct
MRI: Magnetic resonance imaging
PE: Percent edema
PEM: Percent edema mapping
PHS: Percent hemorrhage per slice
PIS: Percent infarct per slice
SI: Signal intensity
TCM: Tissue characterization mapping
TE: Time of echo
TTC: Triphenyl tetrazolium chloride
